# Associations of sperm DNA fragmentation with lifestyle factors and semen parameters of Saudi men and its impact on ICSI outcome

**DOI:** 10.1186/s12958-018-0369-3

**Published:** 2018-05-19

**Authors:** Basmah Al Omrani, Nadia Al Eisa, Murid Javed, Maher Al Ghedan, Hamoud Al Matrafi, Hamad Al Sufyan

**Affiliations:** 1 Zoology Department, King Saud University, Riyadh, Saudi Arabia; 2 Assisted Reproductive Technology Laboratories, Thuriah Medical Center, Riyadh, Saudi Arabia; 3 Genetics Laboratory, Thuriah Medical Center, Riyadh, Saudi Arabia; 4 Urology and Andrology Unit, Thuriah Medical Center, Riyadh, Saudi Arabia

**Keywords:** Sperm DNA fragmentation, Body mass index, ICSI, Infertility, Male factor, Smoking

## Abstract

**Background:**

Male factor infertility is quite common as 30–50% of infertility cases are due to sperm defects. The high sperm DNA fragmentation is one of the causes of male factor infertility. Many factors cause sperm DNA fragmentation and could be testicular or post-testicular. The purpose of this study was to assess relationships among sperm DNA fragmentation, lifestyle factors and semen values of Saudi men and to determine impact of sperm DNA fragmentation on ICSI cycle outcome.

**Methods:**

The duration of this study was from January 2015 to June 2016. The cases with female factor infertility were excluded. In total 94 couples were selected for investigation. The study parameters were male age, body mass index, smoking, semen values, % sperm DNA fragmentation, fertilization rate and pregnancy outcome. The ICSI procedure was performed in all patients per standard protocol. The semen samples were grouped based on % sperm DNA fragmentation into < 15%, 15–30 and > 30% which corresponded to low, moderate and high sperm DNA fragmentation, respectively.

**Results:**

There was no difference in ICSI outcome in low and moderate sperm DNA fragmentation, however, in high sperm DNA fragmentation no patient achieved pregnancy. In this study, 53.19% Saudi men had low, 32.98% moderate and 13.83% high DFI. Semen volume, sperm morphology and fertilization rate did not show any correlation trend with DNA fragmentation, however, sperm concentration and motility were negatively correlated in all DFI categories. The BMI was positively correlated in moderate DFI category and smoking was positively correlated with low DFI category. The age was positively correlated in moderate and high DFI categories.

**Conclusions:**

The results of this study indicated that 14% Saudi men had high DNA fragmentation. The BMI was positively correlated in moderate DFI category and smoking was positively correlated with low DFI category. The age was positively correlated in moderate and high DFI categories.

## Background

Infertility is failure to achieve a clinical pregnancy after 12 months or more of regular unprotected sexual intercourse [1]. It is a common problem, with recent publications quoting a 9 to 18% prevalence in the general population [2]. It may be due to male or female factors or unexplained where after an elaborate workup no apparent reason for infertility is found [3]. About 30–50% of infertility cases are attributed to sperm defects [4, 5]. Defective sperm conditions include very low sperm concentration, inadequate sperm motility and morphological abnormalities. The percentage of cells exhibiting fragmented DNA is represented by the DNA fragmentation index (DFI) [6]. The infertile males are found to have a higher percentage of sperm with defective DNA than fertile controls [7–9]. Therefore, DFI is recommended as an appealing fertility predictive element [10–12].

The causes of sperm DNA damage are numerous, of complex nature and could be testicular or post-testicular [13]. These may include defects in spermatogenesis (e.g., genetic or developmental abnormalities) and testicular or post-testicular injury (e.g., gonadotoxins, hyperthermia, oxidants, and endocrine abnormalities). It has been suggested that protamine deficiency (with consequent aberrant chromatin remodeling), reactive oxygen species and abortive apoptosis may be responsible for sperm DNA damage [14].

Lifestyle factors like increased body mass index (BMI) [15], scrotal hyperthermia [16, 17] and smoking [18] increase DFI. An increased risk of cancer in offspring from fathers with increased level of DFI because of smoking has been reported [19].

Intracytoplasmic sperm injection (ICSI) made a paradigm shift of male infertility treatment. It is recommended even in couples with high level of DFI. The Sperm Chromatin Dispersion (SCD) assay is a validated procedure for assessment of DFI [20]. The DFI has been demonstrated to affect naturally induced pregnancy [21, 22] or pregnancy through ART. Furthermore, the high level of DFI (≥ 27%) has been shown to reduce fertility after ART procedure [23]. The objectives of this study were to determine the level of DFI in Saudi men, to see its impact on ICSI outcome and to evaluate its correlations with lifestyle factors (age, body mass index and smoking) and semen parameters.

## Methods

We retrospectively conducted a systematic review of data from 94 Saudi men tested for sperm DNA fragmentation by SCD assay before undergoing ICSI from Jan 2015 to June 2016 at an infertility clinic in Riyadh, Saudi Arabia. The cases with female factor infertility were excluded. In total 94 couples were selected for investigation. The study parameters were sperm DFI, semen values, male age, BMI, smoking, fertilization and pregnancy rate. The semen samples were divided into following categories based on DFI: < 15%, 15–30% and > 30, which corresponded to low, moderate and high DFI. Thuriah institutional review board approved this study.

### Semen analysis

The patients were instructed to abstain for 3–5 days before providing semen sample by masturbation. After complete liquefaction, semen volume (mL), sperm concentration (10^6^/mL), motility (%) and presence of round sperm (10^6^/ml) were determined. The percent morphology was assessed from a stained smear under 100× oil immersion objective by analysing 200 spermatozoa. The sample was considered normal if it had 1.5–6 ml volume, ≥ 15 million/mL sperm concentration, ≥40% progressive motility and ≥ 4% normal morphology.

### Sperm chromatin dispersion assay

The SCD assay was conducted using Halosperm G2® kit (Halotech®, Spain). At least 300 spermatozoa were scored under an inverted bright-field microscope for each sample. The normal sperm without fragmented DNA showed big or medium size halo and the sperm with fragmented or degraded DNA showed no halo.

### Ovarian stimulation, ICSI and embryo culture

Ovarian stimulation in female partner was achieved using agonist or antagonist protocol. Oocytes were collected 36 h post hCG injection. Oocyte denudation and ICSI were performed per standard procedure [24]. On the day of egg collection, the male partner was asked to produce semen sample which was processed for ICSI. The fertilization was checked 16–18 h post-ICSI. Presence of 2 pronuclei and two polar bodies were recognized as normal fertilization. Oocytes without obvious pronuclei were considered unfertilized. Oocytes with a single pronucleus or more than two pronuclei were considered abnormal fertilization and not included in the study. Embryos were cultured in an atmosphere of 6% CO_2_ in air. Morphological assessment of each embryo was performed daily based on % cytoplasmic fragmentation. Embryos were graded on a scale of 1 to 5 where 1 was considered the best grade with 10% or less fragmentation. Embryo transfer was performed on day 3. Per our clinic policy; 2 embryos were transferred in patients less than 35 yrs., 3 embryos in patients from 36 to 39 yrs. and 4 embryos in patients 40 yrs. or more. The extra embryos were cryopreserved for subsequent embryo transfers. Biochemical pregnancy rate was calculated based on βhCG test 14 days post oocyte retrieval.

### Statistical analysis

The data were analyzed by Statistical Package for Social Sciences (SPSS), Version 23. The main dependent variable in this study was ICSI cycle outcome (successful or unsuccessful). Descriptive statistics were used to calculate mean, medians, maximum and minimum for age, male BMI, semen volume, sperm concentration, sperm motility and morphology. The DFI was categorized into < 15%, 15–30% and > 30, which corresponded to low, moderate and high DFI. The independent t-test was used to detect the difference between two independent groups of ICSI, while Mann-Whitney test was used to assess the significant differences of non-parametric continuous variables (such as semen parameters). Chi-square test was used to assess the significant difference between categorical variables. Pearson’s correlation was used to assess the strength and significance of association between parametric variables, while Spearman’s correlations were used in non-parametric variables. The degree of association was represented by the correlation coefficient and a statistically significant difference was detected when *P* < 0.05.

## Results

From 94 men, 53.19% had low, 32.98% moderate and 13.83% high DFI. The Mean ± SD values for low, moderate and high groups were; 10.46 ± 3.31, 20.80 ± 4.68 and 38.54 ± 10.14%, respectively (Table 1). The distribution of DFI in Saudi sub-fertile men undergoing successful and unsuccessful ICSI treatment is presented in Table 2. The patients in successful group were those who achieved pregnancy in ICSI cycle. The number of embryos transferred in each group were similar. The indication was male factor infertility. There was no significant difference in ICSI outcomes in low (*P* = 0.597) and moderate (*P* = 0.235) DFI categories. In the high DFI category, no patient achieved pregnancy. There were non significant differences between successful and non-successful ICSI groups in regards to the semen parameters. Similarly, there were non significant differences between these two ICSI groups in regards to the age, BMI, and smoking (Table 3).

**Table 1:** Sperm DNA fragmentation categories in Saudi sub-fertile men undergoing ICSI (*n* = 94)

Category	Number (%)	Mean ± SD	Median	Range
Low DFI (< 15%)	50 (53.19)	10.46 ± 3.31	10.94	2.57–14.90
Moderate DFI (15–30%)	31 (32.98)	20.80 ± 4.68	19.80	15.28–29.73
High DFI (> 30%)	13 (13.83)	38.54 ± 10.14	34.43	31.16–67.75

Values are given as number, Mean ± SD, Median and Range

**Table 2:** The distribution of DFI in Saudi sub-fertile men undergoing successful and unsuccessful ICSI treatment (n = 94)

Category	Values in Successful ICSI			Values in Unsuccessful ICSI			*P* Value
	No.	Mean ± SD	Median	No.	Mean ± SD	Median	
DFI < 15%	7	9.83 ± 3.58	10.67	43	10.55 ± 3.29	11.21	0.597
DFI 15–30%	11	19.09 ± 3.78	17.47	20	21.73 ± 4.93	21.75	0.135
DFI > 30%	0	0	0	13	38.54 ± 10.14	34.43	–

Values are given as Mean ± SD and Median. The independent t-test and Chi square were used to compare groups

**Table 3:** The distribution of social and semen characteristics of Saudi sub-fertile men undergoing successful or unsuccessful ICSI treatment

Parameter	Values in Successful ICSI			Values in Unsuccessful ICSI			*P* Value
	No.	Mean ± SD	Range	No.	Mean ± SD	Range	
Male age 23–36 yrs	12	32.75 ± 2.80	28–36	41	32.56 ± 2.67	25–36	0.83
Male age 37–57 yrs	6	42.17 ± 3.19	37–45	35	44.43 ± 5.39	37–55	0.32
BMI = < 30 Kg/m^2^	17	22.65 ± 1.93	19.4–26.7	64	23.89 ± 2.61	19–30	0.48
BMI = > 30 Kg/m^2^	1	32.0		12	32.44 ± 2.35	30–36	–
Smoker	8			34			0.078
Non-smoker	10			42			
Semen Variables							
Volume (mL)	18	2.72 ± 1.15	0.5–5	79	2.86 ± 1.13	0.2–6	0.154
Conc. (10^6^/mL)	18	67.94 ± 74.64	18–350	79	64.13 ± 43.61	15–200	0.628
Prog motility (%)	18	50.16 ± 10.55	40–80	79	49.32 ± 9.19	38–76	0.776
Abn. Morphology (%)	18	94.11 ± 3.32	88–98	79	89.63 ± 11.09	15–98	0.095

The correlations between DFI categories, semen parameters and lifestyle factors are given in Table 4. Semen volume, sperm morphology and fertilization rate did not show any correlation trend with DNA fragmentation; however, sperm concentration and motility were negatively correlated in all DFI categories. The BMI was positively correlated in moderate DFI category and smoking was positively correlated with low DFI category. The age was positively correlated in moderate and high DFI categories.

**Table 4:** The correlations between sperm DFI, semen parameters, fertilization rate and lifestyle factors of the sub fertile men

	DFI% (*P* value)		
Characteristics	< 15	15–30	> 30
Semen Volume (mL)	−0.06 (0.68)	0.209 (0.258)	0.18 (0.556)
Sperm Con. (10^6^/mL)	−0.02 (0.88)	−0.133 (0.475)	− 0.009 (0.976)
Progressive Motility (%)	−0.36 (0.008)^**^	− 0.334 (0.06)	−0.334 (0.264)
Sperm Morphology (%)	−0.096 (0.509)	−0.018 (0.925)	0.198 (0.517)
Fertilization Rate (%)	−0.022 (0.878)	0.05 (0.790)	0.497 (0.084)
Age (Yrs.)	−0.273 (0.366)	0.603 (0.001^)**^	0.548 (0.001^)**^
BMI	0.21 (0.167)	0.42 (0.041)^*^	0.561 (0.073)
Smoking	0.292 (0.040)^*^	0.035 (0.851)	0.340 (0.256)

*P < 0.05

*P < 0.01

## Discussion

In this study, the lowest DFI value was 2.57% and the highest 67.75% in 94 Saudi men tested. In 53% men, the DFI value was 10.46 ± 3.31%. In the moderate and high DFI groups, these values were 20.80 ± 4.68% and 38.54 ± 10.14%, respectively. In the high DFI group no pregnancy was achieved. The DFI has been related to fertility potential [21]. The DFI values are significantly higher in infertile as compared to those in fertile group [25]. A significant inverse correlation was established between DNA fragmentation and sperm concentration, total count, progressive motility (rapid and total) and normal morphology in subfertile and infertile men (*p* < 0.05) as compared to in fertile group [26] Normozoospermic men exhibited lower levels of DNA fragmentation than non-normozoospermic men. Therefore, it was suggested that DNA fragmentation testing and traditional semen analysis should be considered as complementary diagnostic tools in a comprehensive evaluation of male infertility [27].

In our study, the mean DFI value for high DFI group was 38.54% using the SCD assay. The DFI of ≥30% by SCD is considered of poor fertility potential. The other most commonly used techniques to assess sperm DNA integrity are the TUNEL, Comet and Sperm Chromatin Structural Assay (SCSA). A threshold value of 30% DFI was reported for SCSA for IVF/ICSI cases and 20% for the TUNEL assay, although IVF and ICSI term pregnancies have been reported even with semen samples with much higher DFI values [28]. The oocytes and early embryos have the ability to repair sperm DNA damage, with the effect of such damage being dependent on the extent of chromatin damage and the capacity of oocytes to make repairs. This suggests that when sperm has extensively damaged DNA, the capacity of the oocyte to repair damaged DNA might be exceeded and high DFI > 30% may be incompatible with pregnancy [29] in couples with poor oocyte quality.

In the present study, fertilization rate was not different in DFI categories of Saudi men. This finding is in agreement with other studies [30–32]. However, one study found significant association between DFI and fertilization rate [10] which could most likely be due to differences in the oocyte quality because the number of metaphase II oocytes and embryo quality are important factors affecting ICSI outcome. In this study, female age was 33 ± 5.7 yrs. The number of mature oocytes was 10.6 ± 7.5 and oocyte quality was similar in all groups as cases with female factor infertility were excluded.

The sperm DNA fragmentation has been attributed to a variety of intrinsic and extrinsic factors which could be of genetic or environmental origin. Majority of marriages in Saudi population happen in closely related individuals thereby causing some genetic concerns. Among the genetic factors, variations and polymorphisms in genes playing roles in genome integrity have been implicated in an increased risk of sperm DNA fragmentation. The chromosomal structural rearrangements, such as reciprocal translocations are associated with increased DNA damage in infertile men [33]. Analysis of genetic reasons of DNA fragmentation is beyond the scope of this study. A future study addressing this concern is direly needed.

Human sperm DNA is tightly packed. The condensation of nuclear chromatin takes place during the last stages of spermiogenesis and epididymal transport. A few important biochemical changes take place such as the replacement of lysine-rich histones with arginine-rich protamines. At the same time, bisulfidic bonds are formed between cysteine residues. In these ways, the nuclear chromatin is tightly packed to protect it from natural and chemical damaging factors. Abnormalities in sperm chromatin condensation may results in increased sperm DNA fragmentation [34].

This study found that age was significantly positively correlated with moderate and high DFI. This is consistent with an earlier study [35], which aimed to assess the outcomes of ICSI in patients with high DNA fragmentation. It found that male with high and moderate DFI were significantly older than those with the low DFI. This is in disagreement with another study [29] in which there was no significant difference when DFI in males above and below the median age was compared. This non-significant result could be attributed to the life style and young age of included males where maximum age was 48 years, while in the current study the male age reached 55 years.

Among the environmental factors, BMI, smoking and heat to the scrotum are major factors causing DNA fragmentation. In the present study, BMI was significantly correlated with the moderate and total DFI categories. This is in agreement with a study conducted in USA [36], where multiple linear regression detected a significant association between obesity and sperm DNA fragmentation. It found men with BMI higher than 25 to have less DNA integrity, thus, patients should be advised to reduce their body weight in order to achieve maximum possible fertility. This is inconsistent with the findings of a study conducted in Czech [37], where no significant association between BMI and DFI was reported.

The smoking was significantly correlated with each of the high and total DFI categories. Smoking can affect the sperm by losing their ability to fight off free oxygen radicals in the seminal fluid which make the sperm more sensitive to oxidative stress. Therefore, the increases in the free radicals in the seminal fluid affect DFI, motility and fertilization. [29].

About 15% of infertile Saudi men had normal semen parameters despite high DFI; therefore, testing DFI in addition to other semen parameters is beneficial [38] as the DFI evaluation may reveal a hidden abnormality in apparently normal sperm parameters [39].

## Conclusions

In summary, results of this study indicated that 14% Saudi men had high DNA fragmentation. The BMI was positively correlated in moderate DFI category and smoking was positively correlated with low DFI category. The age was positively correlated in moderate and high DFI categories.

## Abbreviations

ART: Assisted reproductive technology
BMI: Body mass index
DFI: DNA fragmentation index
DNA: Deoxyribonucleic acid
hCG: Human chorionic gonadotropin
ICSI: Intracytoplasmic sperm injection
SCD: Sperm chromatin dispersion
SPSS: Statistical package for social sciences
